# Research Progress in Anti-Inflammatory Bioactive Substances Derived from Marine Microorganisms, Sponges, Algae, and Corals

**DOI:** 10.3390/md19100572

**Published:** 2021-10-14

**Authors:** Chao-Qun Li, Qin-Yuan Ma, Xiu-Zhen Gao, Xuan Wang, Bei-Li Zhang

**Affiliations:** 1 School of Life Sciences and Medicine, Shandong University of Technology, Zibo 255000, China; lcqhaida@163.com (C.-Q.L.); Qyma@sdut.edu.cn (Q.-Y.M.); gaoxz@sdut.edu.cn (X.-Z.G.); 2 Key Laboratory of Mariculture (Ministry of Education), Fisheries College, Ocean University of China, Qingdao 266003, China; xuanwang@ouc.edu.cn; 3 Key Laboratory of Aquaculture Nutrition and Feed, Ministry of Agriculture, Ocean University of China, Qingdao 266003, China

**Keywords:** anti-inflammatory activity, inflammatory pathways, natural product, marine bacteria and fungi, marine algae, sponge, coral

## Abstract

Inflammation is the body’s defense reaction in response to stimulations and is the basis of various physiological and pathological processes. However, chronic inflammation is undesirable and closely related to the occurrence and development of diseases. The ocean gives birth to unique and diverse bioactive substances, which have gained special attention and been a focus for anti-inflammatory drug development. So far, numerous promising bioactive substances have been obtained from various marine organisms such as marine bacteria and fungi, sponges, algae, and coral. This review covers 71 bioactive substances described during 2015–2020, including the structures (65 of which), species sources, evaluation models and anti-inflammatory activities of these substances. This review aims to provide some reference for the research progress of marine-organism-derived anti-inflammatory metabolites and give more research impetus for their conversion to novel anti-inflammatory drugs.

## 1. Introduction

Inflammation is a kind of defensive response when the body is affected by various inflammatory factors or local injuries, and it is an important protective mechanism of the biological body [1]. Inflammation usually helps maintain the body’s normal function and promotes repair of damaged tissue to reduce the effect of external stimuli on the body [2,3]. However, an abnormal and excessive inflammatory response can also damage the body’s health and even endanger life [4,5,6]. For instance, the recent SARS-CoV-2 can stimulate the innate immune system, and cause cytokine storms and acute inflammatory responses, which rapidly cause multiple organ failures [7,8]. Steroidal and nonsteroidal anti-inflammatory drugs are clinically applied to cure inflammatory disorders, but long-term use of them is often accompanied by significant side effects [9]. The exploration of safe and effective anti-inflammatory drugs has always been a hotspot of biomedical research.

The ocean is where life is born and nurtured. It covers about 70% of the earth’s surface and 90% of the biosphere. The ocean has special physical and chemical conditions, including high salinity and weak alkalinity; the depths encompass an environment that is dark, cold, subject to high pressures, and presents many other complex characteristics [10]. To better to adapt to such an extreme environment, marine organisms have formed unique genetic systems and biosynthetic pathways and produced novel bioactive metabolites which constitute a huge natural active compound library [11]. For decades, researchers have isolated and purified numerous bioactive products with anti-inflammatory activity from a variety of marine organisms, however, only a few have been approved for clinical trial and even fewer have reached the market [12]. Here, we summarize those promising anti-inflammatory natural products from marine organisms (marine bacteria and fungi, sponges, algae, and coral) and their anti-inflammatory mechanisms, to help researchers to understand the latest research progress in relation to marine anti-inflammatory natural products.

## 2. Inflammatory Pathways and Evaluation Model of Anti-Inflammatory Activity

### 2.1. Inflammatory Pathways

Multiple signaling pathways, including nuclear factor-κB (NF-κB), Janus kinases/signal transducers and activators of transcription (JAK-STAT) and mitogen-activated protein kinase (MAPK) are involved in the regulation of inflammatory response, and play an essential role in a series of physiological and pathological processes in the body.

NF-κB signaling pathway is a classical pathway in inflammation regulation [13]. NF-κB is an important transcriptional regulator in cells, usually in the inactivated form of p50–p65 heterodimer that binds to its inhibitor kappa B (IκB) [14]. After stimulation by inducers, phosphorylation, and proteolysis of IκBα enhance the translocation of NF-κB into the nucleus, where it binds to specific κB sites on DNA to regulate target gene transcription [15]. Activation of NF-κB increases the expression of downstream inflammatory mediators, including pro-inflammatory cytokines (interleukin-1β (IL-1β), IL-6, tumor necrosis factor α (TNFα), etc.), key pro-inflammatory enzymes (inducible nitric oxide synthase (iNOS) and cyclooxygenase-2 (COX-2)), and their derivatives (nitric oxide (NO) and prostaglandin E_2_ (PGE_2_)) [16,17]. Meanwhile, inflammatory mediators such as pro-inflammatory factors induced by NF-κB can in turn activate NF-κB, creating a vicious cycle that amplifies the initial inflammatory response [18].

The JAK-STAT pathway, as a cytokine signaling transduction pathway, has recently attracted much attention. When cytokines bind to cell surface receptors, the receptor molecules dimerize and promote the polymerization and phosphorylation of JAKs. Activated JAKs can bind to the Src homology-2 domain of STATs, which is phosphorylated and activated, eventually entering the nucleus in the form of homologous or heterodimer to initiate the transcription of target genes [19]. Studies indicated that the JAK-STAT signaling pathway is closely associated with the inflammatory differentiation of macrophages [20,21,22]. Interferon-γ (IFN-γ), interleukin and other inflammatory factors can promote the activation of the JAK-STAT signaling pathway, exert signal transduction and transcriptional activation functions, and then affect the M1/M2 type differentiation and inflammatory direction of macrophages [20,23].

MAPK is a type of serine/threonine protein kinases widely distributed in mammals, which can be activated by a three-level kinase cascade process. Extracellular signals stimulate receptors located on the cell membrane to activate MAPKKK, the activated MAPKKK further activates MAPKK, then the activated MAPKK activates MAPK [24]. The transduction process of MAPK signaling mainly consists of three pathways: the c-Jun N-terminal kinase (JNK) pathway, the p38MAPK pathway, and the extracellular regulated protein kinases (ERK) pathway [25,26]. The JNK and p38MAPK pathways can be activated by lipopolysaccharide (LPS), IL-1, TNFα, and other factors [27].

### 2.2. Evaluation Model of Anti-Inflammatory Activity

It is important to select an appropriate model to preliminarily evaluate the activity and the mechanism of anti-inflammatory drugs. The production of pro-inflammatory cytokines by immune cells is a key step in establishing and maintaining an inflammatory response, so it is regarded as the main target of anti-inflammatory intervention [28,29]. The inflammatory models established by macrophages and neutrophils (the main sites of inflammatory response) are the most commonly used and most effective means to assess the anti-inflammatory activity of drug molecules [30,31,32]. Specifically, in vitro anti-inflammatory activity can be evaluated by measuring NO release, mRNA expression and/or production of inflammatory modulators (IL-1/2/5/6/8/10/12/25, TNFα, PGE_2_, etc.), and expressions of key protein (iNOS, COX-2, etc.) in macrophage cells RAW264.7 or THP-1 and other cell types (splenocytes, BV2 microglia, dendritic cells (DCs), etc.) induced by LPS, ovalbumin, or IFN-γ [33,34]. Researchers also stimulated neutrophils with LPS and assessed the anti-inflammatory activity of the drug molecule by examining its influence on superoxide anion production or elastase secretion [12].

Mice or rats are commonly chosen as experimental animals to build the in vivo inflammation model. Xylene, arachidonic acid, or croton oil can induce acute exudative inflammatory edema in the ear of experimental animals [35,36]. Intra-plantar use of carrageenan in the hind paws of the experimental animals can also induce acute inflammation and the anti-inflammatory activity of drug molecule can be assessed by measuring improvements at the inflammatory site [37]. Furthermore, dextran sulphate sodium (DSS) and 2,4,6-trinitrobenzene sulfonic acid (TNBS) are frequently employed to induce colitis in mice. The typical characteristics of mouse colitis are shortened mucosal folds, swelling of the lamina propria and subepithelial mucosa, and severe infiltration of various inflammatory cells, increased mRNA expression of proinflammatory cytokines, increased intestinal mucosal permeability, etc. [38,39] The anti-inflammatory activity of drug molecules can be assessed by measuring the changes in such indicators. Additionally, the zebrafish is an attractive in vivo model due to its small size, high fecundity and full annotation of genome. Several chemical-based inflammation models of zebrafish induced by LPS, DSS, TNBS or CuSO_4_ have been established and the anti-inflammatory activity of drug molecule can be evaluated through the suppression of various inflammatory symptoms [40,41].

## 3. Anti-Inflammatory Bioactive Substances Derived from Marine Organisms

### 3.1. Marine Bacteria and Fungi

Marine bacteria and fungi are an important part of marine ecosystems; they can survive and reproduce continuously in low-pressure, low-temperature, or other extreme environments such as those under high pressure, high temperature, and high salinity. Compared with terrestrial microorganisms, marine bacteria and fungi are more likely to produce natural secondary metabolites with novel structures and high activities. Marine bacteria and fungi have been the frontier of drug discovery and numerous bioactive compounds have been obtained from them [42,43]. The anti-inflammatory bioactive substances derived from marine bacteria and fungi in this review were shown in Table 1.

#### 3.1.1. Anti-Inflammatory Peptides from Marine Bacteria and Fungi

**Figure 1:**
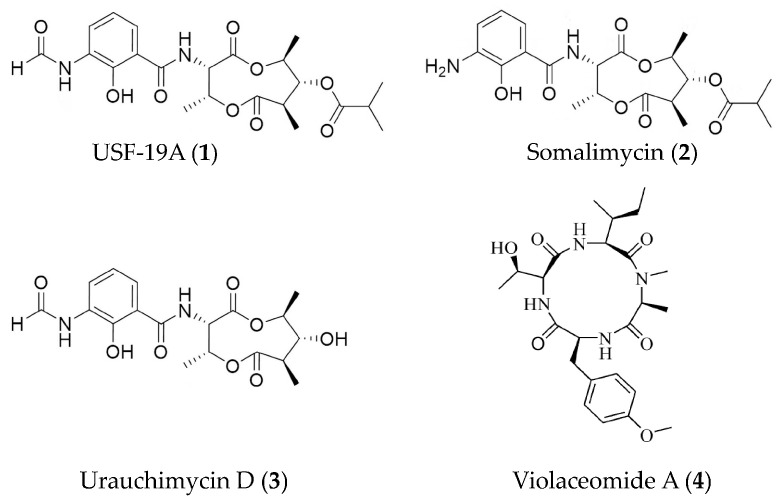
Structures of anti-inflammatory peptides from marine bacteria and fungi.

Among various microorganisms, marine actinomycetes have long been one of the favored strains in research related to drug development. Antimycin-type depsipeptides USF-19A (**1**), somalimycin (**2**), and urauchimycin D (**3**) (Figure 1) from a mutant strain of *Streptomyces somaliensis* SCSIO ZH66 can suppress the IL-5 production in splenocytes induced by ovalbumin in mouse [44]. Compound **1** demonstrated strong inhibitory activity with an IC_50_ value of 0.57 μM, while compounds **2** and **3** displayed mild effects (>10 μM). Moreover, the three depsipeptides exhibited very weak cytotoxicity against human umbilical vein endothelial cells with LD_50_ values of 62.6, 34.6, and 192.9 μM. The new cyclic peptide, violaceomide A (**4**) (Figure 1), from a marine sponge-derived fungus *Aspergillus violaceofuscus* showed inhibitory activity on the mRNA expression of IL-10 in the LPS-stimulated THP-1 cells (a human acute monocytic leukemia cell line) with inhibitory rate of 84.3% at 10 μM [45].

#### 3.1.2. Anti-Inflammatory Polyketides from Marine Bacteria and Fungi

**Figure 2:**
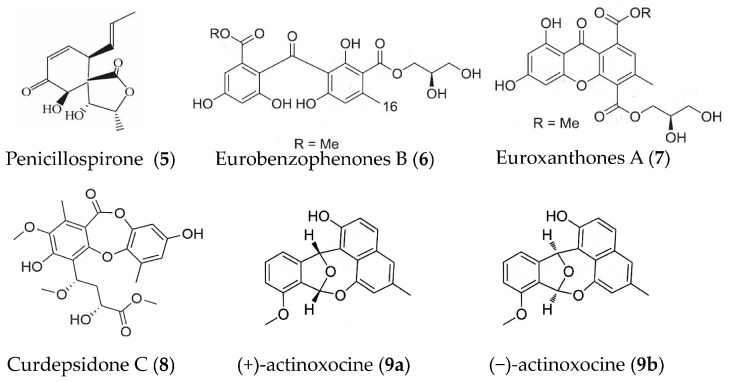
Structures of anti-inflammatory polyketides from marine bacteria and fungi.

A new polyketide-type metabolite, penicillospirone (**5**) (Figure 2) was isolated from the EtOAc extract of a marine-derived fungus *Penicillium* sp. SF-5292 and demonstrated inhibitory activity against the overproduction of NO and PGE_2_ in LPS-induced RAW264.7 macrophages and BV2 microglia, which was correlated with the suppressive effect against over-expression of iNOS and COX-2. It could also inhibit the production of pro-inflammatory cytokines including TNFα, IL-1β, IL-6, and IL-12. Further study confirmed that the anti-inflammatory effect of compound **5** was mediated through the negative regulation of the NF-κB pathway [46]. Six new polyketide derivatives, eurobenzophenones A-C, euroxanthones A-B, and (+)1-O-demethylvariecolorquinones A were isolated from the sponge associated fungus *Aspergillus europaeus*. Eurobenzophenones B (**6**) and euroxanthones A (**7**) (Figure 2) significantly down-regulated NF-κB in LPS-induced SW480 cells (human colon carcinoma cell line) with weak inhibition on NO production in LPS induced BV2 cells [47]. Curdepsidone C (**8**) (Figure 2) was obtained from fungus *Curvularia* sp. IFB-Z10 (isolated from the intestine of a white croaker) and showed remarkable anti-inflammatory activity against IL-1β release, with an IC_50_ value of 7.47 ± 0.35 μM in *Propionibacterium acnes*-induced THP-1cells [48]. (+)- and (−)-actinoxocine (**9a, 9b**) (Figure 2) were isolated from a marine-derived *Streptomyces* sp. and showed inhibition on TNFα protein release in LPS- and Pam3CSK4-induced RAW 264.7 mouse macrophages, respectively [49].

#### 3.1.3. Other Anti-Inflammatory Substances from Marine Bacteria and Fungi

**Figure 3:**
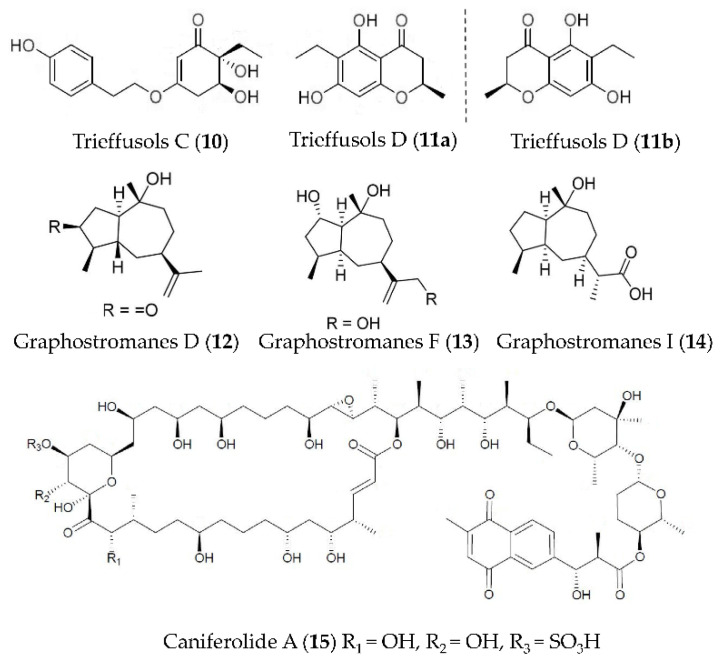
Structures of anti-inflammatory phenol derivatives, guaianes, and macrolides from marine bacteria and fungi.

Two new highly substituted phenol derivatives, trieffusols C (**10**) and D (**11**) (Figure 3), were isolated from the extract of deep-sea-sediment-derived *Trichobotrys effuse* FS524 and showed the inhibition of NO production in murine macrophages with IC_50_ values ranging from 51.9 to 55.9 μM [49]. New guaianes, including graphostromanes D (**12**), F (**13**) and I (**14**) (Figure 3), were isolated from *Graphostroma* sp. MCCC 3A00421 derived from a hydrothermal sulfide deposit. Compound **13** can inhibit the NO release in RAW264.7 macrophages induced by LPS with an IC_50_ value of 14.2 μM—stronger than that of aminoguanidine—a positive control with an IC_50_ value of 23.4 μM. Compounds **12** and **14** showed weak anti-inflammatory activities, with IC_50_ values of 72.9 and 88.2 μM respectively [50]. The macrolide caniferolide A (**15**) (Figure 3) from *Streptomyces caniferus* could block NFκBp65 translocation to the nucleus and showed inhibition on the production of pro-inflammatory cytokines (IL-1β, IL-6 and TNFα), the release of NO, and the activities of iNOS, JNK and p38 in LPS induced BV2 microglial cells [51].

**Figure 4:**
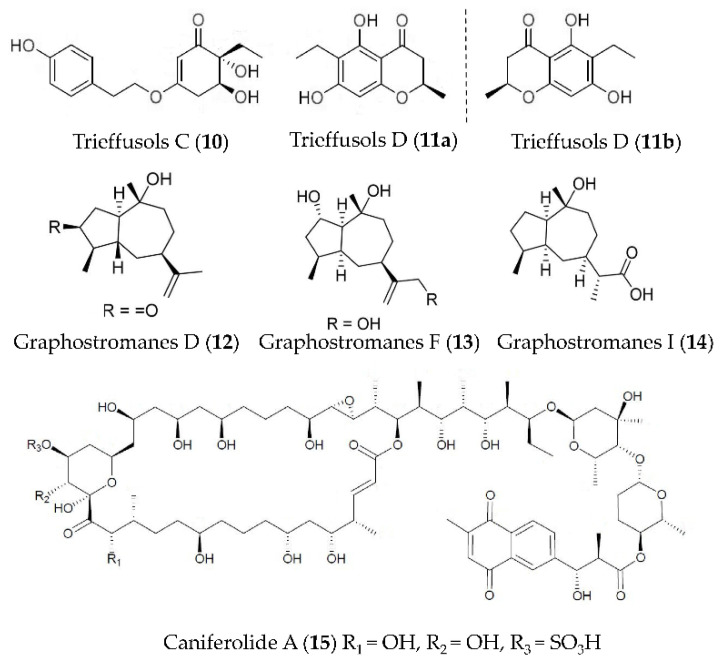
Structures of anti-inflammatory phenazine and saphenic acid derivatives from marine bacteria and fungi.

Phenazine derivatives, 6-[1-(2-aminobenzoyloxy)ethyl]-1-phenazinecarboxylic acid (**16**), saphenol (**17**), (R)-saphenic acid (**18**), phenazine-1-carboxylic acid (**19**), 6-(1-hydroxyehtyl)phenazine-1-carboxylic acid (**20**), and 6-acetyl-phenazine-1-carboxylic acid (**21**) (Figure 4), were isolated from a marine fungus *Cystobasidium larynges* IV17-028. They showed moderate inhibition on NO production in mouse macrophage RAW264.7 cells induced by LPS at 30 μg/mL [53].

**Figure 5:**
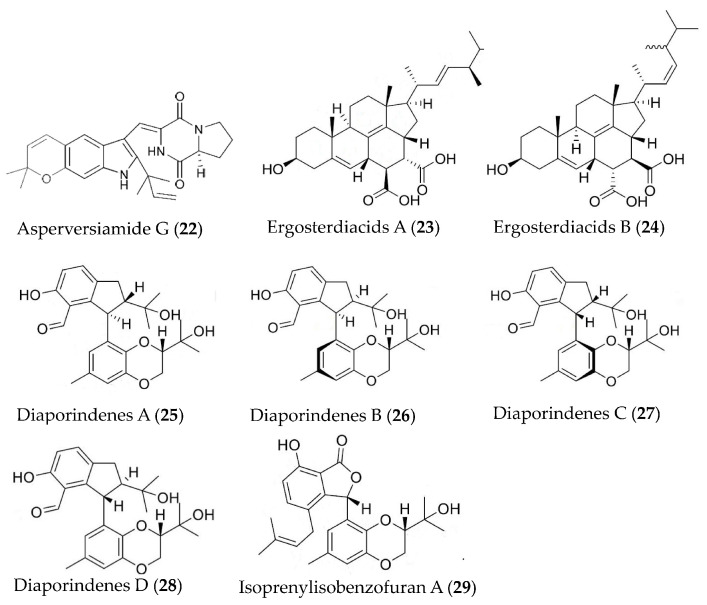
Structures of anti-inflammatory alkaloids and steroids from marine bacteria and fungi.

Asperversiamide G (**22**) (Figure 5) was isolated from the marine-derived fungus *Aspergillus versicolor* and showed inhibition against iNOS with an IC50 value of 5.39 μM [54]. Two naturally Diels-Alder additive steroids, ergosterdiacids A (**23**) and B (**24**) (Figure 5), were isolated from mangrove-derived fungus *Aspergillus* sp. and displayed strong in vitro anti-inflammatory activities against the NO production at 4.5 and 3.6 μM, respectively [55]. Diaporindenes A–D (**25**–**28**), four unusual 2, 3-dihydro-1H-indene isomers and a novel isoprenylisobenzofuran A (**29**), were separated from *Diaporthe* sp. SYSU-HQ3. Compounds **25**–**29** (Figure 5) exhibited remarkable inhibitory effects against NO production with IC_50_ values from 4.2 to 9.0 μM [56].

### 3.2. Marine Sponges

Sponges, as the most primitive multicellular animals, have been living in the ocean for around 600 million years. To date, more than 10,000 types of sponges have been discovered, accounting for about ^1^/_15_ of all marine animal species. Sponge has become one of the most abundant marine organisms in the discovery of marine active substances and represents an excellent resource for marine drug exploitation. To date, approximately 84 anti-inflammatory compounds have been isolated from marine sponges [9]. The anti-inflammatory bioactive substances derived from sponges in this review were shown in Table 2.

#### 3.2.1. Anti-Inflammatory Peptides from Marine Sponge

**Figure 6:**
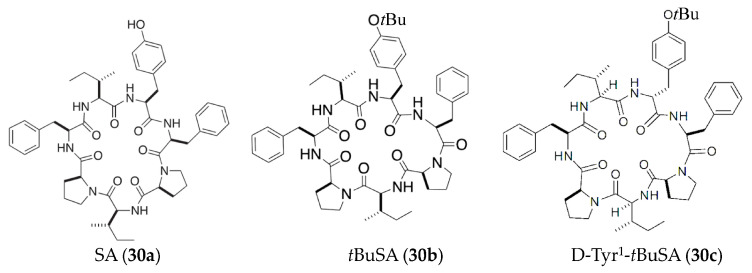
Structures of anti-inflammatory peptides from marine sponge.

Stylissatin A (SA) (**30a**) (Figure 6), a proline-rich cyclic heptapeptide isolated from the marine sponge *Stylissa massa*, could suppress NO production in LPS-induced murine RAW264.7 macrophage cells (EC_50_ = 87 μM) [57]. Further study reported that the activities of a *tert*butyl ether analogue of SA (*t*BuSA, **30b**) (Figure 6) were approximately six times stronger than natural SA (**30a**) (EC_50_ = 12 μM) with little cytotoxicity at up to 200 μM [58]. A recent study also indicated that a SA derivative D-Tyr^1^-*t*BuSA (**30c**) (Figure 6) could inhibit the production of IL-6 and TNFα (EC_50_ = 1.4 and 5.9 μM, respectively) and the expression of iNOS (EC_50_ = 20 μM) in LPS-stimulated RAW264.7 cells [59].

#### 3.2.2. Anti-Inflammatory Terpenoids from Marine Sponge

**Figure 7:**
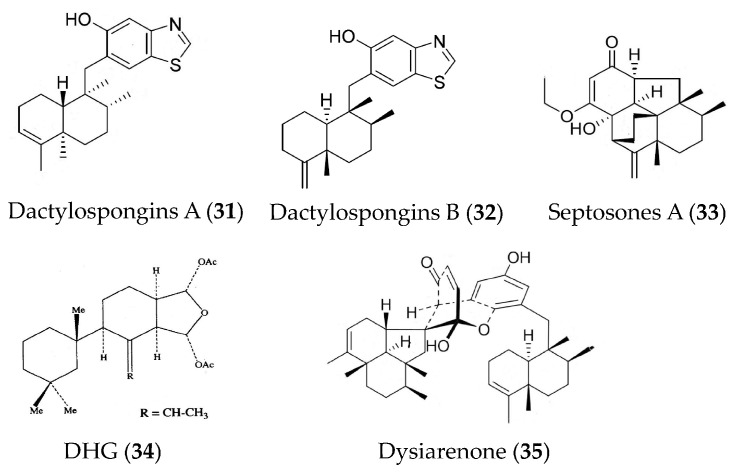
Structures of anti-inflammatory terpenoids from marine sponge.

Dactylospongins A (**31**) and B (**32**) (Figure 7) are new sesquiterpenoids isolated from the marine sponge *Dactylospongia* sp. collected from the South China Sea. They can inhibit the production of various cytokines (IL-6, IL-1β, IL-8, and PGE_2_) in LPS-stimulated THP-1 cells; however, neither showed significant effects on the production of monocyte chemotactic protein 1 and TNFα [60]. Three meroterpenoids (septosones A–C) were isolated from the marine sponge *Dysidea septosa*. Septosone A (**33**) (Figure 7) indicated in vivo anti-inflammatory activity that it could alleviate migration and reduce the number of macrophages surrounding the neuromast in CuSO_4_-induced transgenic zebrafish in a dose-dependent manner and could inhibit TNFα-induced NF-κB activation in human HEK-293T cells with an IC_50_ value of 6.8 μM [61]. The 9,11-dihydrogracilin A (DHG, **34**) (Figure 7) extracted from Antarctic marine sponge *Dendrilla membranosa* showed remarkable immunomodulatory and anti-inflammatory effects. An in vitro study indicated that compound **34** could induce apoptosis of human peripheral blood mononuclear cells and down-regulate the phosphorylation of NF-κB, STAT, and ERK at late time points. Meanwhile, compound **34** induced the down-regulation of IL-6 and IL-10. Compound **34** also reduced the growth, viability, and migration of HaCaT cells (human keratinocyte cell line). An in vivo study showed that topical use of compound **34** significantly decreased mouse ear edema [62]. The dysiarenone (**35**) (Figure 7) isolated from the marine sponge *Dysidea arenaria* exhibited inhibitory activities against COX-2 expression and PGE_2_ production in LPS-stimulated RAW264.7 macrophages [63].

#### 3.2.3. Other Anti-Inflammatory Substances from Marine Sponge

**Figure 8:**
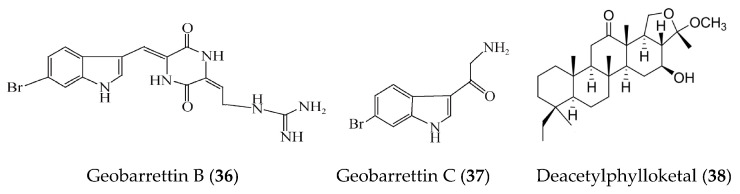
Structures of anti-inflammatory alkaloids and phylloketal derivative from marine sponge.

Geobarrettin B (**36**) and C (**37**) (Figure 8) are new bromoindole alkaloids isolated from the sub-Arctic sponge *Geodia barretti*. Compounds **36** and **37** reduced IL-12p40 secretion of DCs, but compound **37** concomitantly increased IL-10 production. Maturing DCs treated with compound **36** or **37** before co-culturing with allogeneic CD4⁺ T cells were found to reduce the IFN-γ secretion, indicating potential for the treatment of TH1-type inflammation [64]. A new phylloketal derivative, deacetylphylloketal (**38**) (Figure 8), was obtained from the sponge genus *Phyllospongia* and could suppress the production and/or gene expression of NO, PGE_2_, IL-6, IL-1β, and TNFα. Compound **38** could also suppress the expression of iNOS and COX-2 in a co-culture system that consisted of human epithelial Caco-2 cells and PMA-differentiated THP-1 macrophage cells [65].

### 3.3. Marine Algae

Marine algae are the oldest existing lower cryptogamous plants, with a wide variety of species (about 30,000 known to date). At present, four groups of seaweeds have been extensively exploited, including blue algae, red algae, brown algae, and green algae. Marine algae are known to be a rich source of bioactive metabolites and interesting pharmacological substances. The search for bioactive metabolites from seaweed has been very active [66]. The anti-inflammatory bioactive substances derived from marine algae in this review were shown in Table 3.

#### 3.3.1. Anti-Inflammatory Peptides and Proteins from Marine Algae

Marine lectins are glycoproteins or peptides that bind to specific mono or oligosaccharides, which can promote cell recognition and adhesion, and some of them also showed strong anti-inflammatory activity. A lectin from the red marine alga *Solieria filiformis* reduced neutrophil migration in a peritonitis model and decreased paw edema induced by carrageenan, dextran, and serotonin with no signs of systemic damage in mice [67]. The anti-inflammatory mechanism of a lectin from the green seaweed *Caulerpa cupressoides* var. *lycopodium* was investigated and showed that it decreased the carrageenan-induced rat paw edema and neutrophilic infiltration at 0.1, 1 or 10 mg/kg, and inhibited the expression of IL-1β, IL-6, TNFα and COX-2 at 1 mg/kg [68].

#### 3.3.2. Anti-Inflammatory Polysaccharides from Marine Algae

Polysaccharides are the main components of marine algae, which have attracted much attention because of their various health benefits [79]. Certain marine algal polysaccharides showed significant anti-inflammatory activities, which have been confirmed by several inflammatory models. A fucoidan from brown algae inhibited Poly(I:C) (a TLR3 agonist that mimics viral RNA)-induced expression of some cytokines (IL-1α, IL-1β, TNFα, and IL-6) and PGE_2_ but did not change the IL-12/25 production, indicating that locally applied fucoidan might suppress airway inflammation in viral infections [69]. The high molecular weight fucoidan from *Fucus vesiculosus* L. (Mw 735 kDa, sulfate content 27%, fucose 73.5 mol%, glucose 11.8 mol%, galactose 3.7 mol%, xylose 6.6 mol%, mannose 0.2 mol%, and arabinose 0.2 mol%) showed remarkable anti-inflammatory activity through the inhibition of COX-1/2, hyaluronidase and MAPK p38 [70]. The purified fucoidan fraction from *Turbinaria ornate* (sulfate content 27%) displayed anti-inflammatory potential that could suppress NO production (IC_50_ = 30.83 ± 1.02 μg·mL^−1^) and dose-dependently reduce iNOS, COX-2, and pro-inflammatory cytokines including PGE_2_ levels in LPS-induced RAW264.7 macrophages and inhibit the production of NO and ROS in LPS-induced zebrafish embryo [71]. *Turbinaria ornata*, a brown alga of the Sargassaceae family, is rich in bioactive molecules with various biological activities. The sulfated polysaccharide isolated from *T. ornate* could significantly reduce the paw volume and arthritic score in complete Freund’s adjuvant induced arthritis in rats. Interestingly, the sulfated polysaccharide could alleviate inflammation and bone damage at a low dose (5 mg/kg), indicating its potential in the management of chronic inflammatory diseases [72].

#### 3.3.3. Other Anti-Inflammatory Substances from Marine Algae

**Figure 9:**
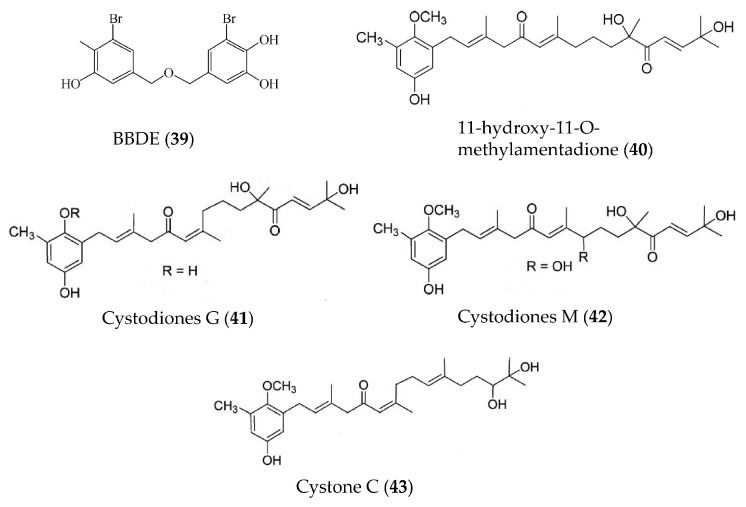
Structures of anti-inflammatory bromophenols and terpenoids from marine algae.

A bromophenol, bis (3-bromo-4,5-dihydroxybenzyl) ether (BBDE, **39**) (Figure 9), isolated from the red alga *Polysiphonia morrowii* displayed anti-inflammatory activity by reducing inflammatory mediators, including NO, PGE_2_, iNOS, COX2, and pro-inflammatory cytokines (TNFα, IL-1β, and IL-6) in LPS-induced macrophage cells [73]. Further studies have indicated that BBDE could suppress LPS-induced inflammation by inhibiting the reactive oxygen species (ROS)-mediated ERK signaling pathway [73]. A meroditerpene, 11-hydroxy-1′-O-methylamentadione (**40**) (Figure 9), from the brown alga *Cystoseira usneoides* displayed anti-inflammatory activity through increasing mucus production, reducing myeloperoxidase activity and decreasing inflammatory mediators (TNFα, IL-1β, IL-10, iNOS and COX2) [74]. Three new meroditerpenoids, cystodiones G (**41**) and M (**42**) and cystone C (**43**) (Figure 9), were also isolated from *Cystoseira* *usneoides* and showed significant inhibition on TNFα production in LPS-stimulated THP-1 human macrophages [75].

**Figure 10:**
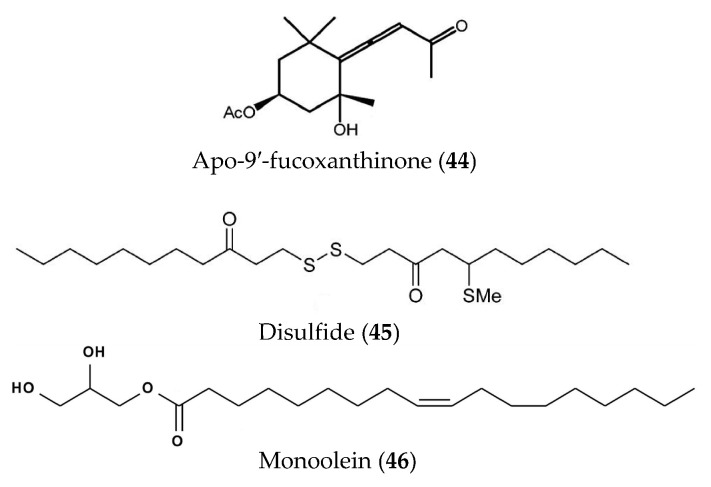
Structures of apo-9′-fucoxanthinone, disulfide and monoolein from marine algae.

Apo-9′-fucoxanthinone (**44**) (Figure 10) derived from *Undariopsis* *peterseniana* showed strong anti-inflammatory activity both in vitro and in vivo. Compound **44** showed significant inhibition of NO, PGE_2_, iNOS and COX-2, and pro-inflammatory cytokines (TNFα, IL-6, and IL-1β) in LPS-stimulated RAW 264.7 cells and can relieve inflammatory stress and suppress the expression of COX-2 and iNOS in LPS-stimulated zebrafish embryos [76]. A new disulfide (**45**) (Figure 10) was obtained from the brown alga *Dictyopteris* *membranacea* and displayed strong inhibition of NO production in LPS-induced RAW264.7 macrophages [77]. The anti-inflammatory activity and underlying mechanism of monoolein (**46**) (Figure 10) isolated from *Ishige Sinicola* were studied and showed that it could inhibit the production of IL-12 p40, IL-6 and TNFα, and suppress the activation of MAPK and NF-κB pathways through the inhibition of the phosphorylation of p38, ERK1/2, JNK1/2, and IκBα [78].

### 3.4. Marine Corals

Coral is a large group of invertebrates belonging to the phylum Cnidaria, which is a low primitive organism with a wide distribution and with a wide variety of species (about 7000 known at time of writing). Coral is a marine biological resource that can be used extensively, in particular, soft corals and Gorgonians have been ranked highly with regard to the discovery of bioactive metabolites with potential pharmaceutical applications [80]. In recent decades, researchers have isolated a variety of bioactive compounds from soft corals and Gorgonians, including terpenoids, sterols, alkaloids, and long-chain fatty acids, some of which have novel structures and significant physiological activities such as antivirus, anti-inflammatory, antibacterial, anti-tumor, and immunosuppressive activities [81]. The anti-inflammatory bioactive substances derived from corals in this review were shown in Table 4.

#### 3.4.1. Anti-Inflammatory Terpenoids from Marine Corals

**Figure 11:**
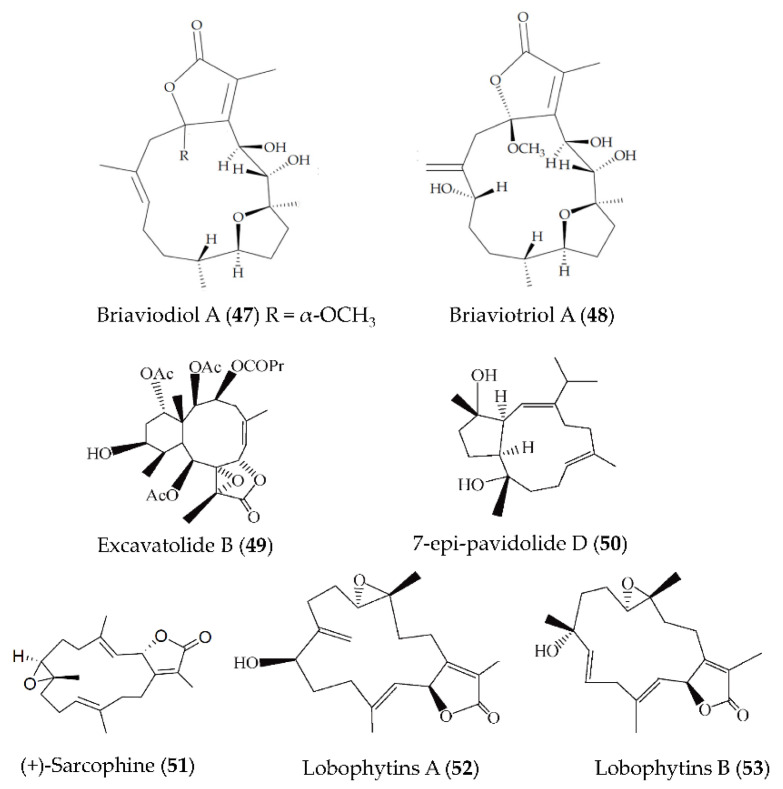
Structures of anti-inflammatory terpenoids from marine corals.

A new furanocembranoid–briaviotriol A (**47**)–along with a known analogue (briaviodiol A, **48**) (Figure 11), were obtained from *Briareum violaceum*. Compounds **47** and **48** showed inhibition on LPS-induced iNOS release in macrophages with inhibition rates of 67.7 and 61.9%, respectively (at a dose of 10 μM) [82]. A natural diterpene product, excavatolide B (**49**) (Figure 11), isolated from gorgonian *Briareum excavatum*, could significantly inhibit the mRNA expression of the proinflammatory mediators, including iNOS and COX-2 in LPS-induced RAW 264.7 macrophages [83]. Furthermore, compound **49** could attenuate carrageenan-induced paw edema by inhibiting the expression of iNOS and immune cell infiltration [83]. A new capnosane-based diterpenoid, 7-epi-pavidolide D (**50**) (Figure 11), was obtained from the marine soft coral *Klyxum flaccidum*, and could suppress superoxide anion generation and elastase release in the N-formyl-methionyl-leucyl-phenylalanine/cytochalasin B (fMLP/CB)-induced human neutrophils [84]. A diterpenoid, (+)-sarcophine (**51**) (Figure 11), isolated from a soft coral *Sarcophyton stellatum* showed anti-inflammatory activity by reducing the expressions of COX-2 and iNOS in LPS-stimulated mouse RAW 264.7 macrophage cells [85]. Two cembrane-type diterpenoids (lobophytins A (**52**) and B (**53**)) (Figure 11) were isolated from the soft coral *Lobophytum sarcophytoides* and exerted inhibitory effects on NO production in RAW264.7 cells with IC_50_ values of 26.7 and 17.6 µM, respectively [86].

**Figure 12:**
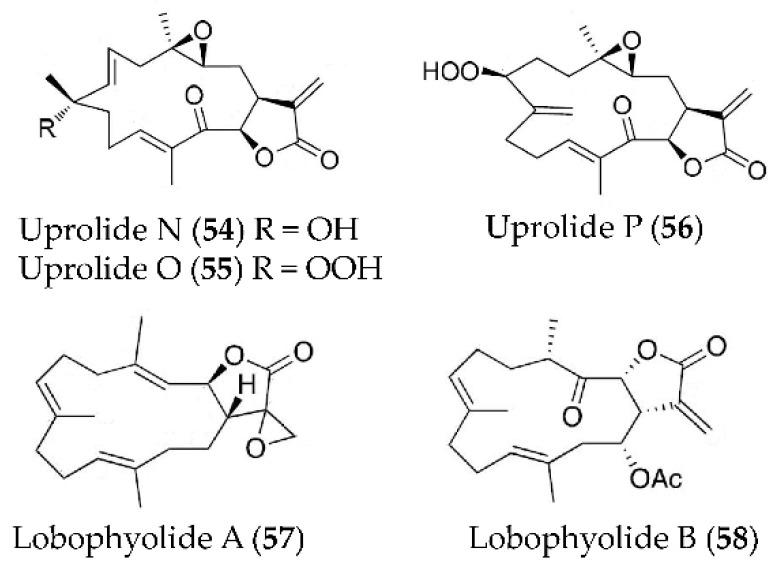
Structures of anti-inflammatory terpenoids from marine corals.

Three new diterpenes, uprolide N (**54**), uprolide O (**55**) and uprolide P (**56**) (Figure 12), were isolated from *Eunicea succinea* and showed remarkable inhibitory effect on the production of TNFα and IL-6 in LPS-induced peritoneal macrophages [87]. Two new cembrane-type diterpenoids, lobophyolide A (**57**) and B (**58**) (Figure 12), were isolated from a wild-type soft coral *Lobophytum crissum* and could suppress IL-12 release and NO production in LPS-activated DCs [88].

#### 3.4.2. Other Anti-Inflammatory Substances from Marine Corals

**Figure 13:**
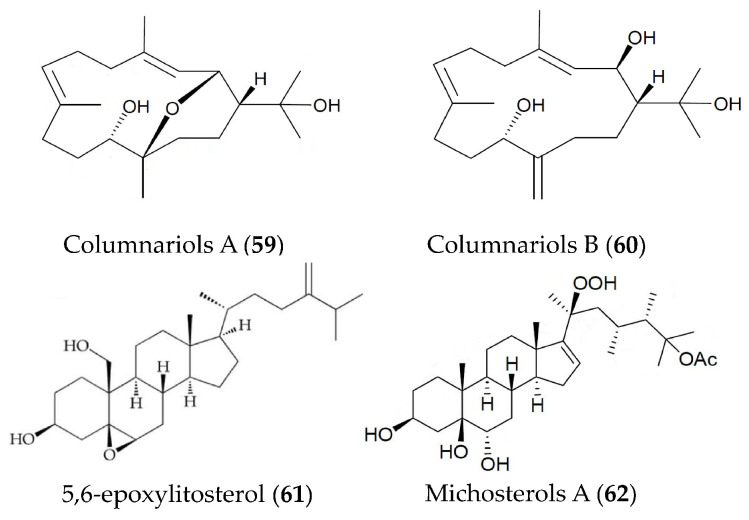
Structures of anti-inflammatory cembranes, sterols and polyoxygenated steroids from marine corals.

Two new cembranes (columnariols A (**59**) and B (**60**)) (Figure 13), were isolated from the soft coral *Nephthea columnaris* and play a significant inhibitory role in the accumulation of the pro-inflammatory iNOS and COX-2 protein in LPS-stimulated RAW264.7 macrophage cells. Compound **58** showed moderate cytotoxicity against human prostatic carcinoma tumor cells with an IC_50_ value of 9.80 μg/mL [89]. A sterol (5,6-epoxylitosterol, **61**) (Figure 13) obtained from the octocoral *Nephthea columnaris* showed anti-inflammatory activity via suppressing superoxide anion production and elastase secretion in fMet-Leu-Phe/Cytochalastin B-induced human neutrophils [90]. A new polyoxygenated steroid (michosterols A, **62**) (Figure 13) isolated from the ethyl acetate extract of the soft coral *Lobophytum michaelae* also showed superior anti-inflammatory activity via suppressing superoxide anion generation and elastase release in fMLP/CB-stimulated human neutrophils [91].

**Figure 14:**
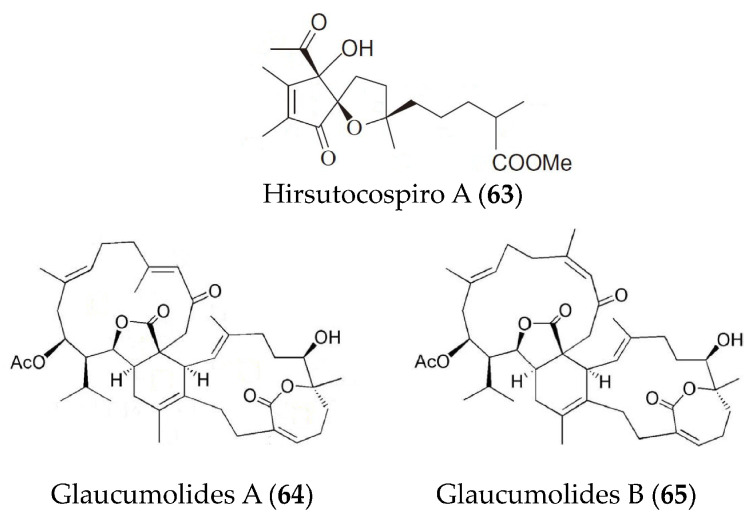
Structures of anti-inflammatory hirsutocospiro A and glaucumolides A and B from marine corals.

A new tocopherol-derived metabolite, hirsutocospiro A (**63**) (Figure 14), was obtained from *Cladiella hirsute* and displayed strong anti-inflammatory activity in fMLF/CB-induced human neutrophils [92]. Glaucumolides A (**64**) and B (**65**) (Figure 14) from *Sarcophyton glaucum* exhibited strong inhibition of superoxide anion generation and elastase release in fMLP/CB-stimulated human neutrophils and showed inhibition on the iNOS and COX-2 expression in LPS-induced RAW264.7 macrophages [93].

## 4. Conclusions and Research Prospects

Inflammation, especially chronic inflammation, is a crucial contributor to the development of various human diseases. Regulation of inflammation to maintain its normal level is a key step in the treatment of related diseases. Although existing steroidal and non-steroidal anti-inflammatory drugs contribute a great deal, long-term use often causes adverse effects, including gastrointestinal discomfort, liver and kidney dysfunction, damage to the cardiovascular system, endocrine system, and so on. Marine organisms offer hope for the development of safe and effective new anti-inflammatory drugs. This review was conducted to provide reference for the research progress and give more impetus for the conversion of marine-organism-derived natural products to anti-inflammatory drugs. The Web of Science (WOS), PubMed, ScienceDirect, SpringerLink, and ACS databases were used for the preparation of the review, and some keywords such as “anti-inflammatory activity, natural product, marine bacteria and fungi, sponges, algae, and coral, etc.” were used for the search of relevant information. Finally, 71 bioactive substances described during 2015–2020 were presented, including the structures (65 of which), species sources, evaluation models and anti-inflammatory activities. Furthermore, some limitations could be obtained in this review: although a wide coverage was expected to be achieved, it’s extremely difficult to cover all the relevant literatures in view of the huge richness and diversity of marine organisms and their natural products; furthermore, a certain degree of randomness indeed exists for the presentation of the relevant literatures in the research field.

Research into anti-inflammatory drugs derived from marine organisms started relatively late, but it has developed rapidly. As reviewed here, many anti-inflammatory substances have been obtained from a wide variety of marine organisms, including marine bacteria and fungi, sponges, algae, and corals. Preliminary studies have been conducted on their anti-inflammatory activities and mechanisms. Of course, we also need to be aware that the development and application of marine drugs still face many challenges. First, the extreme environment in which marine organisms live is difficult to simulate in the laboratory, which makes it extremely difficult to cultivate marine organisms and obtain large quantities of their active ingredients. Furthermore, the clinical effect and market application of some marine active substances remain uncertain due to their own limitations. For instance, although bioactive peptides have many well-known advantages, their clinical effects are often unable to match experimental results from the laboratory due to their complex structure, low concentration of active components, and closed N-terminal. Finally, a thorough safety assessment is crucial, as small differences in the amount used may lead to a shift in the role of the active products between poison and therapeutic.

In future, we should try to investigate two aspects of this research: (1) take the isolated anti-inflammatory active substances from marine organisms as lead compounds, conduct functional modifications thereof, and study their structure-function relationship, so as to screen anti-inflammatory drugs with better efficacy; (2) strengthen resource integration, establish a comprehensive and efficient technological platform integrating detection, fermentation culture, separation and purification, functional modification, and effect evaluation, thus improving the efficiency of the development and application of new anti-inflammatory drugs. The ocean is a vast treasure trove, and there are still many bioactive compounds that have not been exploited. More extensive and in-depth studies should be conducted to find other, potentially valuable, marine drugs.

## Figures and Tables

**Table 1:** Anti-inflammatory bioactive substances derived from marine bacteria and fungi.

Bioactive Substances	Species	Model	Activities	Reference
USF-19A (**1**), somalimycin (**2**), and urauchimycin D (**3**)	*Streptomyces somaliensis* SCSIO ZH66	ovalbumin-stimulated mouse splenocytes	against IL-5 with IC_50_ values of 0.57 μM, > 10 μM and > 10 μM	[44]
Violaceomide A (**4**)	*Aspergillus violaceofuscus*	LPS-stimulated THP-1 cells	against mRNA expression of IL-10 with inhibitory rate of 84.3% at 10 μM	[45]
Penicillospirone (**5**)	*Penicillium* sp. SF-5292	LPS-induced RAW264.7 macrophages and BV2 microglia	against the production of NO, PGE_2_, TNFα, IL-1β, IL-6, and IL-12	[46]
Eurobenzophenones B (**6**) euroxanthones A (**7**)	*Aspergillus europaeus*	LPS induced BV2 microglia	against NO at 10 μM	[47]
Curdepsidone C (**8**)	*Curvularia* sp. IFB-Z10	*Propionibacterium acnes*-induced THP-1cells	against IL-1β release with an IC_50_ value of 7.47 ± 0.35 μM	[48]
(+)- and (−)-actinoxocine (**9a**, **9b**)	*Streptomyces* sp.	LPS- and Pam3CSK4-induced RAW 264.7 mouse macrophages	against TNFα protein release	[49]
Trieffusols C and D (**10**, **11**)	*Trichobotrys effuse* FS524	LPS-induced RAW264.7 macrophages	against NO with IC_50_ values ranging from 51.9 to 55.9 μM	[50]
Graphostromanes D, F and I (**12**–**14**)	*Graphostroma* sp. MCCC 3A00421	LPS-induced RAW264.7 macrophages	against NO with IC_50_ values of 14.2, 72.9 and 88.2 μM	[51]
Caniferolide A (**15**)	*Streptomyces caniferus*	LPS induced BV2 microglial cells	against NFκBp65 translocation to the nucleus, the production of IL-1β, IL-6 and TNFα, the release of NO, and the activities of iNOS, JNK and p38	[52]
6-[1-(2-aminobenzoyloxy) ethyl]-1-Phenazinecarboxylic acid (**16**), Saphenol (**17**), (R)-saphenic acid (**18**), Phenazine-1-carboxylic acid (**19**), 6-(1-hydroxyehtyl) phenazine-1-carboxylic acid (**20**), 6-acetyl-phenazine-1-carboxylic acid (**21**)	*Cystobasidium larynges* IV17-028	LPS-induced RAW264.7 macrophages	against NO production at 30 μg/mL	[53]
Asperversiamide G (**22**)	*Aspergillus versicolor*	LPS-induced RAW264.7 macrophages	against iNOS with an IC_50_ value of 5.39 μM	[54]
Ergosterdiacids A and B (**23**, **24**)	*Aspergillus* sp.	LPS-induced RAW264.7 macrophages	against NO with IC_50_ values of 4.5 and 3.6 μM	[55]
Diaporindenes A-D (**25**–**28**), isoprenylisobenzofuran A (**29**)	*Diaporthe* sp. SYSU-HQ3	LPS-induced RAW264.7 macrophages	against NO with IC_50_ values from 4.2 to 9.0 μM	[56]

**Table 2:** Anti-inflammatory bioactive substances derived from marine sponge.

Bioactive Substances	Species	Model	Activities	Reference
SA and *t*BuSA (**30a**, **30b**)	*Stylissa massa*	LPS-induced RAW264.7 macrophages	against NO with EC_50_ values of 87 μM	[57,58]
D-Tyr^1^-*t*BuSA (**30c**)	*Stylissa massa*	LPS-induced RAW264.7 macrophages	against production of IL-6 and TNFα (EC_50_ = 1.4 and 5.9 μM, respectively) and the expression of iNOS (EC_50_ = 20 μM)	[59]
Dactylospongins A and B (**31**, **32**)	*Dactylospongia* sp.	LPS-stimulated THP-1 cells	against production of IL-6, IL-1β, IL-8, and PGE_2_ with IC_50_ values of 5.1–9.2 μM	[60]
Septosones A (**33**)	*Dysidea septosa*	CuSO_4_-induced zebrafish; human HEK-293T cells	against migration of macrophages surrounding the neuromast; against TNFα-induced NF-κB activation with IC_50_ value of 6.8 μM	[61]
9,11-dihydrogracilin A (DHG, **34**)	*Dendrilla membranosa*	Phytohemagglutinin-activated Human peripheral blood mononuclear cells	against production of IL-6 and IL-10 at 3 μM	[62]
Dysiarenone (**35**)	*Dysidea arenaria*	LPS-induced RAW264.7 macrophages	against COX-2 expression and PGE_2_ production with IC_50_ value of 6.4 μM	[63]
Geobarrettin B and C (**36**, **37**)	*Geodia barretti*	LPS-activated DCs	against secretion of IL-10 with inhibitory rate of 29% and 13% at 10 μg/ml	[64]
Deacetylphylloketal (**38**)	*Phyllospongia* sp.	LPS-induced co-culture system that consisted of human epithelial Caco-2 cells and THP-1 macrophage cells	against production and/or gene expression of NO, PGE_2_, IL-6, IL-1β, and TNFα, iNOS, and COX-2	[65]

**Table 3:** Anti-inflammatory bioactive substances derived from marine algae.

Bioactive Substances	Species	Model	Activities	Reference
Lectin	*Solieria filiformis*	carrageenan-induced peritonitis and paw edema induced by carrageenan, dextran, and serotonin	against neutrophil migration in peritonitis model and decreased paw edema	[67]
Lectin	*Caulerpa cupressoides*	zymosan-induced arthritis of the rat temporomandibular joint	against leukocyte influx and the expression of IL-1β and TNFα at concentrations of 0.1, 1 or 10 mg/kg	[68]
Fucoidan	*Ascophyllum nodosum*	Poly(I:C)-induced human bronchial epithelial cells	against the production of cytokines (IL-1α, IL-1β, TNFα, and IL-6) and PGE_2_ at the concentration of 0.1% (m/v)	[69]
Fucoidan	*Fucus vesiculosus* L.	LPS-induced human mononuclear U937 cells	against COX-1, COX-2 and hyaluronidase activity with IC_50_ values of 27, 4.3 and 2.9 μg/mL, and concentration-dependently inhibit the MAPK p38	[70]
Purified fucoidan fraction	*Turbinaria ornata*	LPS-induced RAW264.7 macrophages and zebrafish embryo	against NO production with IC_50_ value of 30.83 μg/mL and dose-dependently against iNOS, COX-2, and pro-inflammatory cytokines including PGE2 levels; against production of NO and ROS	[71]
Fucoidan like sulphated polysaccharide	*Turbinaria ornata*	Freud’s adjuvant induced mouse arthritis	against inflammation and bone damage at a low dose of 5 mg/kg	[72]
BBDE (**39**)	*Polysiphonia morrowii*	LPS-induced RAW264.7 macrophages	against NO, PGE_2_, iNOS, COX2, and pro-inflammatory cytokines (TNFα, IL-1β, and IL-6) at 2 μM	[73]
11-hydroxy-1′-O-methylamentadione (**40**)	*Cystoseira usneoides*	DSS-induced mouse colitis	Increasing mucus production and against myeloperoxidase activity, production of TNFα, IL-1β and IL-10, and expression of COX-2 and iNOS	[74]
Cystodiones G and M (**41**, **42**), cystone C (**43**)	*Cystoseira usneoides*	LPS-stimulated THP-1 human macrophages	against the production of TNFα at concentrations of 10, 8 and 5 μM	[75]
Apo-9′-fucoxanthinone (**44**)	*Undariopsis peterseniana*	LPS-stimulated RAW 264.7 cells; LPS-stimulated zebrafish embryos	against NO, PGE_2_, iNOS and COX-2, and pro-inflammatory cytokines (TNFα, IL-6, and IL-1β); against inflammatory stress and expression of COX-2 and iNOS	[76]
Disulfide (**45**)	*Dictyopteris membranacea*	LPS-induced RAW264.7 macrophages	against NO with IC_50_ value of 3.8 µM	[77]
Monoolein (**46**)	*Ishige sinicola*	LPS-stimulated primary murine bone marrow-derived dendritic cells	against IL-12 p40, IL-6, and TNFα production with IC_50_ values of 1.69, 6.87, and 5.19 μM; against the activation of MAPK and NF-κB pathways by inhibiting the phosphorylation of p38, ERK1/2, JNK1/2, and IκBα	[78]

**Table 4:** Anti-inflammatory bioactive substances derived from marine corals.

Bioactive Substances	Species	Model	Activities	Reference
Briaviodiol A (**47**) briaviotriol A (**48**)	*Briareum violaceum*	LPS-induced RAW264.7 macrophages	against iNOS release with inhibitory rate of 67.7% and 61.9% at 10 μM	[82]
Excavatolide B (**49**)	*Briareum excavatum*	LPS-induced RAW264.7 macrophages; carrageenan-induced mouse paw edema	against iNOS protein expression at concentrations ranging from 1 to 50 μM and against iNOS protein expression at 50 μM; against edema and redness of hind paws at 15 and 60 mg/kg	[83]
7-epi-pavidolide D (**50**)	*Klyxum flaccidum*	fMLF/CB-induced human neutrophils	against 24.46% of superoxide anion generation and 29.96% of elastase release with IC_50_ > 10 μM	[84]
(+)-Sarcophine (**51**)	*Sarcophyton stellatum*	LPS-induced RAW264.7 macrophages	against iNOS protein expression at 50 and 100 µM, and COX-2 expression at 25–100 µM	[85]
Lobophytins A and B (**52**, **53**)	*Lobophytum sarcophytoides*	LPS-induced RAW264.7 macrophages	against NO with IC_50_ values of 26.7 and 17.6 µM	[86]
Uprolide N, O and P (**54**–**56**)	*Eunicea succinea*	LPS-induced peritoneal macrophages	against TNFα production with IC_50_ values of 1.39, 2.73 and 2.27 µM, and against IL-6 production with IC_50_ values of 3.26, 4.22 and 2.60 µM	[87]
Lobophyolide A and B (**57**, **58**)	*Lobophytum crassum*	LPS-activated DCs	against IL-12 release with inhibitory rate of 93.4% and 93.6% at 50 µg/mL; against NO production with inhibitory rate of 93.5% and 95.9% at 50 µg/mL	[88]
Columnariols A and B (**59**, **60**)	*Nephthea columnaris*	LPS-induced RAW264.7 macrophages	against iNOS and COX-2 protein expressions at 50 µM	[89]
5,6-epoxylitosterol (**61**)	*Nephthea columnaris*	fMet-Leu-Phe/Cytochalastin B induced human neutrophils	against superoxide anions generation and elastase release with IC_50_ values of 4.60 and 3.90 µM	[90]
Michosterols A (**62**)	*Lobophytum michaelae*	fMLF/CB-induced human neutrophils	against superoxide anions generation and elastase release with IC_50_ values of 7.1 and 4.5 µM	[91]
Hirsutocospiro A (**63**)	*Cladiella hirsuta.*	fMLF/CB-induced human neutrophils	against superoxide generation and elastase release with IC_50_ values of 4.1 and 3.7 µM	[92]
Glaucumolides A and B (**64**, **65**)	*Sarcophyton glaucum*	fMLP/CB-stimulated human neutrophils; LPS-induced RAW264.7 macrophages	against superoxide anion generation and elastase release with IC_50_ values of 2.79 and 3.97 µM; against iNOS and COX-2 expression at concentrations of 10 and 20 µM	[93]
